# Efficacy and safety of rituximab in elderly patients with membranous nephropathy

**DOI:** 10.3389/fphar.2023.1323334

**Published:** 2023-12-22

**Authors:** Yanhong Guo, Huayan Zhao, Mingjing Ren, Yulin Wang, Liuwei Wang, Lin Tang

**Affiliations:** ^1^ Department of Nephropathy, The First Affiliated Hospital of Zhengzhou University, Zhengzhou, Henan, China; ^2^ Department of Critical Care Medicine, The First Affiliated Hospital of Zhengzhou University, Zhengzhou, Henan, China

**Keywords:** membranous nephropathy, rituximab, elderly, effectiveness, safety

## Abstract

**Objectives:** Advancing age is a risk factor for treatment-related side effects and mortality in membranous nephropathy (MN) patients treated with traditional immunosuppressive regimens. This study aimed to determine the efficacy and safety of rituximab (RTX) in the treatment of elderly patients with MN.

**Methods:** We performed a single center retrospective review of 37 consecutive MN patients aged 70 and older at the time of RTX infusion. We also enrolled 76 young patients (<70 years old) with MN as the control group. We assessed clinical and laboratory indices, remission rates, and adverse events at RTX infusion, 3 months, and last visit.

**Results:** A total of 37 elderly patients with MN were included, with a median follow-up period of 15.50 (10.00, 24.40) months. Of the 37 patients, 75.68% were male, and mean age was 71.89 ± 2.47 years. At last visit, 7 (18.92%) patients achieved complete remission, and 26 (70.27%) patients achieved complete or partial remission. There were no differences in the complete remission rate and complete or partial remission rate at last visit compared to young patients (26.32% vs*.* 18.92%, *p* = 0.387; 85.53% vs*.* 70.27%, *p* = 0.055). After RTX treatment, three of 6 elderly patients with pneumonia died due to ineffective treatment of the infection in RTX therapy courses. The results of multivariant regression analysis showed that elderly patients have an increased risk of serious infection, compared with patients younger than 70 years (OR = 32.874, 95% CI 1.300–831.490, *p* = 0.034). For each increase of 1 g/L in serum albumin, the risk of serious infection would decrease by 43.2% (OR = 0.568, 95% CI 0.334–0.969, *p* = 0.038).

**Conclusion:** This study demonstrates that RTX is effective in the treatment of elderly patients with MN. However, we also observed a high incidence of infectious complications. Our experience was limited by its retrospective design and relatively small sample size, and further randomized controlled studies with large sample size are needed to confirm our preliminary findings.

## 1 Introduction

Ageing has steadily emerged as a major global public health issue as elderly people continues to rise along with economic development, public health awareness, and medical technology advancements. Recent studies indicated that the incidence rate of membranous nephropathy (MN) has increased in recent years (Hu et al., 2020; Ronco et al., 2021), and it is the most common pathological type of primary nephrotic syndrome in the elderly (Gorsane et al., 2021; He et al., 2023).

Long-term follow-up studies had shown that 35%–40% of patients with MN and massive proteinuria who are not treated may ultimately die or develop end stage renal disease (Ponticelli et al., 1995). However, it is very difficult to treat elderly patients with MN, as they mostly suffer from multiple underlying diseases, poor immunity, higher infection risk, and poor medical compliance (Koratala and Tantravahi, 2018). Therefore, for elderly patients with MN, the advantages and disadvantages should be fully weighed before the use of glucocorticoids and immunosuppressive drug (Abrass, 2003).

Rituximab (RTX), a selective B-cell depleting agent, has a relatively more favorable safety profile (Gauckler et al., 2021). The effectiveness of RTX in the treatment of MN has been investigated in several studies, including randomized controlled trials (Dahan et al., 2017; Fervenza et al., 2019; Scolari et al., 2021). The 2021 KDIGO guidelines also recommended that RTX can be used as a first-line regimen for the treatment of MN (Kidney Disease: Improving Global Outcomes KDIGO Glomerular Diseases Work Group, 2021). The impact of aging on the effectiveness and safety of RTX in MN patients, however, have not been well studied. As a result, we created this retrospective study to assess the effectiveness and safety of RTX in elderly patients with MN.

## 2 Methods

### 2.1 Study design and patients

This was a single center retrospective study. This study comprised a total of 37 elderly patients (aged ≥70 years old) and 76 young patients (aged <70 years old) with MN who were treated with RTX at the First Affiliated Hospital of Zhengzhou University between January 2019 and December 2021.

**FIGURE 1:**
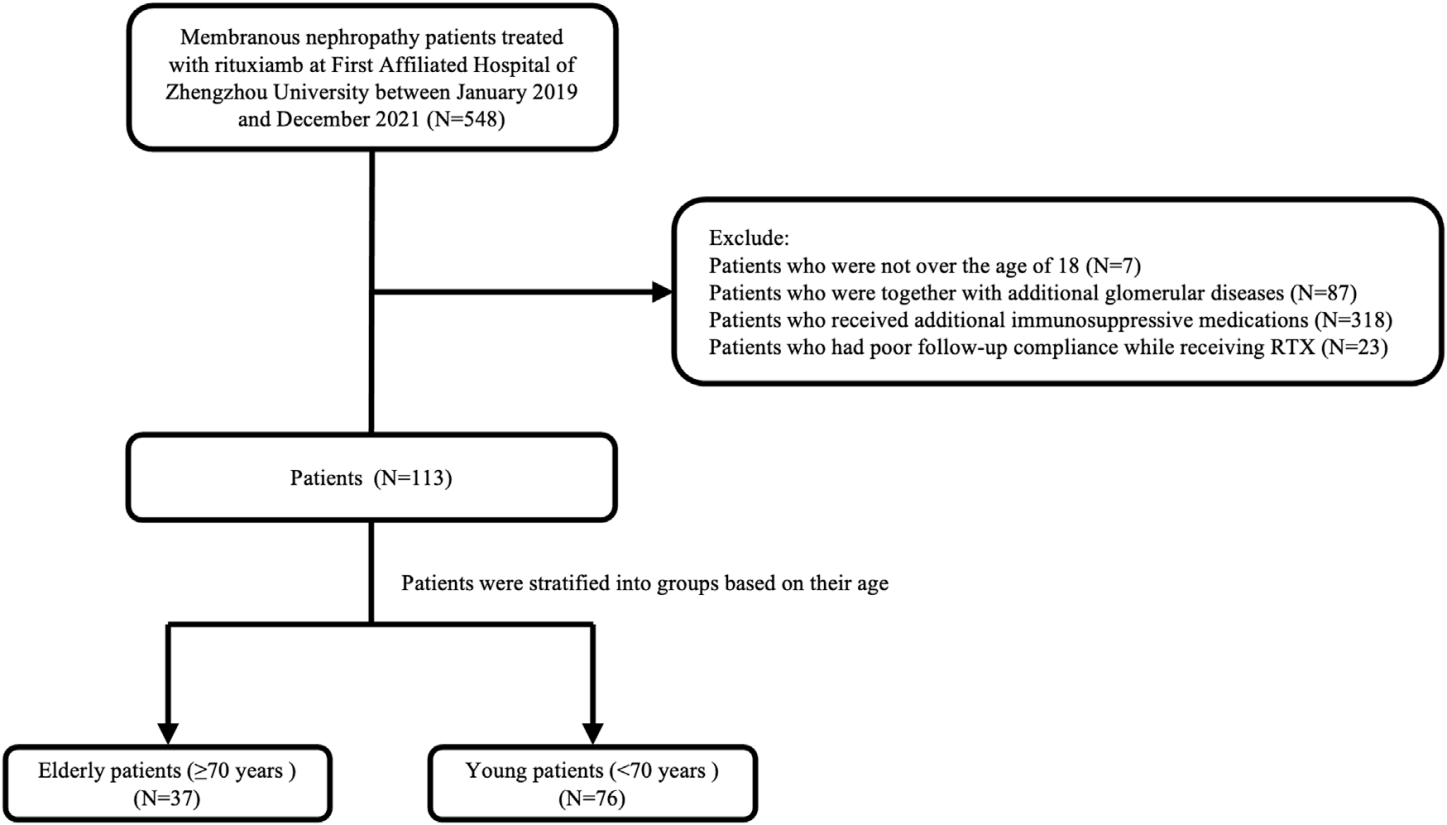
The flowchart of this study.

The following were the exclusion requirements: 1) Patients who had secondary MN or other glomerular diseases; 2) Patients who use glucocorticoids or other immunosuppressants simultaneously; 3) Patients who had poor follow-up compliance while receiving RTX. A study flow chart is shown in Figure 1. The study was approved by the First Affiliated Hospital’s ethical committee at Zhengzhou University.

### 2.2 Immunosuppressive regimen and follow-up

RTX was redissolved in normal saline to a final concentration of 1 mg/mL, and infused at an initial rate of 20 mL/h, and then gradually increased to 100 mL/h according to the tolerance of each patient. The number of RTX infusions, the dose of each injection, and the dosing interval between injections were determined by the treating physician based on immune status, the level of anti-phospholipase A2 receptor (PLA2R) antibody, and CD 19 + B cell counts of patients. All enrolled patients had to be monitored for a minimum of 6 months. When patients received other immunosuppressants or died, follow-up came to an end. To prevent infections, all patients were administered 1 sulfamethoxazole compound tablets (sulfamethoxazole, 0.4 g and trimethoprim, 80 mg), once a day orally after RTX infusion until CD 19 + B cells recover to baseline levels.

General clinical information, including gender, age, blood pressure, BMI (body mass index), and past medication regimens were gathered from medical records. Laboratory findings including hemoglobulin, liver and kidney function tests, blood lipids, 24 h urine protein, anti-PLA2R antibody, and circulating B-cell quantity were collected at the time of RTX infusion and repeated at subsequent 1–3 months intervals after RTX administration. The estimated glomerular filtration rate (eGFR) was calculated using the Chronic Kidney Disease Epidemiology Collaboration algorithm according to age, gender, race, and serum creatinine levels.

### 2.3 Treatment response

According to the 2021 KDIGO recommendations, complete remission was defined as urinary protein excretion <0.3 g/24 h with normal serum albumin and serum creatinine. Partial remission was defined as proteinuria <3.5 g/24 h or a reduction of ≥50% from peak, improvement or normalization of serum albumin, and stable serum creatinine. Immunologic remission was defined as the titer of anti-PLA2R antibody below 2 RU/mL.

### 2.4 Statistical analysis

Data could be expressed as mean ± standard deviation, median, interquartile range, or percentages. Paired sample *t*-test or the Wilcoxon matched pair signed-rank (two samples) test was used for comparison of the results before and after RTX treatment according to the distribution of data. Independent sample t-tests or non-parametric tests are used to compare the differences between elderly patients and young patients, and Mann-Whitney tests are used for non-parametric tests. Differences between categorical variables in the two groups of patients were compared using either the chi-square test or the Fisher exact test. Multiple logistic regression analysis was used to control for confounding variables and identify risk factors that were significantly predictive of nonremission and infections.

## 3 Results

### 3.1 Baseline characteristics at RTX infusion

A total of 37 elderly patients with MN were included in this study with a median follow-up period of 15.50 (10.00, 24.40) months. The demographics and clinical characteristics of those elderly patients at the date of RTX initiation were listed in Table 1. In the group of elderly patients, there were 9 female and 28 male patients with a mean age of 71.89 ± 2.47 years. The median duration of MN since percutaneous renal biopsy was 24.00 months (IQR 5.00–59.00 months). Twenty-five elderly patients had been administered at least one course of immunosuppressant therapy, of which 19 were treated with tacrolimus and 6 were treated with cyclosporine. Sixteen of the 25 elderly patients achieved complete or partial remission during prior therapies. Twenty-eight elderly patients received the 375 mg/m^2^ x4 doses RTX infusion. The other 9 elderly patients received less than 2 g of RTX to achieve immunological remission or B-cell depletion (<5 cells/mm^3^), with 2 elderly patients receiving one dose of 1 g per patient, 1 elderly patient receiving two infusions for a total dose of 1.2 g per patient, and 6 elderly patients receiving three infusions for a total dose of 1.60 ± 0.15 g per patient.

**TABLE 1:** Baseline characteristics of patients with MN.

Variables	Age ≥ 70 years (*n* = 37)	Age < 70 years (*n* = 76)	*p*-valve
Demographics			
Gender (male/female)	28/9	55/21	0.709
Age at the first infusion (years)	71.89 ± 2.47	49.93 ± 10.61	<0.001*
Diabetes mellitus (N, %)	10 (27.03%)	19 (25.00%)	0.817
Hypertension (N, %)	24 (64.86%)	42 (55.26%)	0.331
Disease duration (months)	24.00 (5.00, 59.00)	22.00 (9.50, 36.00)	0.771
Period of follow-up (months)	15.50 (10.00, 24.40)	14.00 (12.00, 18.00)	0.485
Rituximab dose (g)	2.00 (1.90, 2.30)	2.00 (2.00, 2.50)	0.728
BMI (kg/m^2^)	25.27 ± 3.50	25.91 ± 4.11	0.423
BP (mmHg)			
Systolic	131.32 ± 14.88	126.51 ± 10.51	0.083
Diastolic	79.19 ± 11.29	89.76 ± 10.60	0.269
Creatinine (µmol/L)	105.00 (74.00, 133.50)	90.00 (72.00, 144.00)	0.143
eGFR (ml/min/1.73 m^2^)	57.84 (46.33, 81.93)	80.80 (56.72, 103.70)	0.001*
eGFR >90	6	34	0.049*
eGFR 60–89	12	20	
eGFR 30–59	15	18	
eGFR 15–29	3	3	
eGFR<15	1	1	
24 h urine protein (g/d)	5.04 (3.98, 9.22)	6.42 (4.65, 10.985)	0.066
Albumin (g/L)	25.85 ± 6.30	25.57 ± 6.34	0.828
anti-PLA2R-Ab titer (RU/mL)	51.30 (12.50, 107.80)	24.70 (6.06, 67.10)	0.114
PLA2R associated MN (N, %)	34 (91.89)	64 (84.21%)	0.476
Number (%) of patients with, (N, %)			
Initial therapy	12 (32.43%)	30 (39.47%)	0.232
Relapse	16 (43.24%)	37 (48.68%)	
Ineffective	9 (24.32%)	9 (11.84%)	
Number (%) of patients with, (N, %)			
Low-risk	0 (0.00%)	5 (6.58%)	0.323
Intermediate-risk	4 (10.81%)	11 (14.47%)	
High-risk	33 (89.19%)	59 (77.63%)	
Very high-risk	0 (0.00%)	1 (1.32%)	
Previous therapies			
Prednisone + cyclophosphamide, n	0	11	0.107
Prednisone + cyclosporine, n	6	16	
Tacrolimus, n	19	37	
Mycophenolate mofetil, n	0	2	

Data presented as median (first-third interquartile range) or mean ± SD, or number (percentage). *Stands for *p* < 0.05. MN, membranous nephropathy; BP, blood pressure; eGFR, estimated glomerular filtration rate; BMI, body mass index; anti- PLA2R-Ab, anti-phospholipase A2 receptor antibody.

By the time of enrollment, the median level of proteinuria was 5.04 (3.98, 9.22) g/d, and serum creatinine was 105.00 (74.00, 133.50) µmol/L in elderly patients. There were 4 (10.81%) elderly patients who were with eGFR <30 mL/min/1.73 m^2^. There were 34 (91.90%) elderly patients who were positive for anti-PLA2R antibody, and the baseline level of anti-PLA2R antibody titer was 51.30 (12.50, 107.80) RU/mL.

Compared to young patients, eGFR in elderly patients was lower [57.84 (46.33, 81.93) vs*.* 80.80 (56.72, 103.70) mL/min/1.73 m^2^, *p* < 0.001]. However, the level of serum creatinine between the two groups showed no differences [90.00 (72.00, 144.00) vs*.* 105.00 (74.00, 133.50) µmol/L, *p* = 0.143]. Therefore, the significant differences in eGFR might be due to the different age. There was no significant difference between the two groups in other baseline characteristics, including gender, disease duration, period of follow-up, rituximab dose, 24-h urine protein quantification, serum albumin, and the level of anti-PLA2R antibody (Table 1).

### 3.2 Treatment responses

After RTX treatment, at month 3, 2 (5.41%) and 18 (48.65%) elderly patients achieved complete remission and complete or partial remission, respectively. Continued improvements were observed with 7 (18.91%) and 26 (70.27%) elderly patients demonstrating complete remission and complete or partial remission at last visit (Table 2). Compared to young patients, the number of patients with complete remission and complete or partial remission showed no differences at month 3 and last visit (all *p* > 0.05).

**TABLE 2:** Efficacy outcome variables in elderly patients and young patients treated with rituxiamb at month 3 and the last visit.

Variables	Age≥70 years (*n* = 37)	Age<70 years (*n* = 76)	*p*-valve
Characteristics at month 3			
Remission, complete and partial (N, %)	17 (45.95%)	33 (43.42%)	0.800
Remission, complete (N, %)	3 (8.11%)	6 (7.89%)	0.969
24 h urine protein (g/d)	2.87 (1.32, 5.79)	3.13 (1.33, 7.34)	0.390
Albumin (g/L)	27.25 (23.08, 33.18)	33.35 (28.50, 35.55)	0.014*
Serum creatinine (µmol/L)	91.50 (67.75, 114.75)	82.00 (65.75, 119.50)	0.414
eGFR (ml/min/1.73 m^2^)	71.27 (57.03, 84.38)	85.78 (62.50, 104.13)	0.004*
anti-PLA2R-Ab-depleted patients (N, %)	17 (50.00%)	39 (60.94%)	0.298
anti-PLA2R-Ab titer (RU/mL)	3.80 (2.00, 20.65)	2.00 (2.50, 5.95)	0.037*
Characteristics at month last visit			
Remission, complete and partial (N, %)	26 (70.27%)	65 (85.53%)	0.055
Remission, complete (N, %)	7 (18.92%)	20 (26.32%)	0.387
24 h urine protein (g/d)	1.60 (0.49, 3.75)	0.84 (0.30, 2.0)	0.073
Albumin (g/L)	34.90 (28.10, 39.05)	37.10 (34.08, 40.23)	0.061
Serum creatinine (µmol/L)	100.00 (71.00, 115.00)	81.00 (68.00, 106.25)	0.220
eGFR (ml/min/1.73 m^2^)	64.95 (54.10, 85.42)	84.00 (65.74, 102.27)	<0.001*
anti-PLA2R-Ab-depleted patients (N, %)	25 (73.53%)	57 (89.06%)	0.048*
anti- PLA2R-Ab titer (RU/mL)	2.00 (2.00, 2.20)	2.00 (2.00, 2.00)	0.064

Data presented as number (percentage) or median (first-third interquartile range). *Stands for *p* < 0.05. eGFR, estimated glomerular filtration rate; anti- PLA2R-Ab, anti-phospholipase A2 receptor antibody; Complete and partial remissions were defined according to 2021 KDIGO, criteria on the basis of proteinuria.

In the group of elderly patients, the median CD19^+^ B cell count was 220.99/mm^3^ (IQR, 127.00–411.50) at baseline. There was a significant reduction in circulating CD19^+^ B cells at month 3 compared with the baseline level [(1.00 (0.00, 4.00) vs. 220.99 (127.00, 411.50)/mm^3^, *p* < 0.001]. At last visit, CD19^+^ B cell counts had recovered to some extent, and there was still significant difference between the baseline level and that at the end of follow-up [7.00 (1.40, 66.00) vs. 220.99 (127.00, 411.50)/mm^3^, *p* < 0.001]. Anti-PLA2R antibody was detectable in 34 (91.90%) elderly patients at baseline in this study. At month 3 and the last visit, anti-PLA2R antibody titers [3.80 (2.00, 20.65) vs. 51.30 (12.50, 107.80) RU/mL, *p* < 0.001; 2.00 (2.00, 2.20) vs. 51.30 (12.50, 107.80) RU/mL, *p* < 0.001] were lower compared with the baseline levels in elderly patients. Immunologic remission was observed in 17 of 34 (50.00%) and 25 of 34 (73.53%) elderly patients at month 3 and last visit. There were no differences in the level of CD19^+^ B cell count and anti-PLA2R antibody level between elderly patients and young patients at month 3 and last visit (all *p* > 0.05).

**FIGURE 2:**
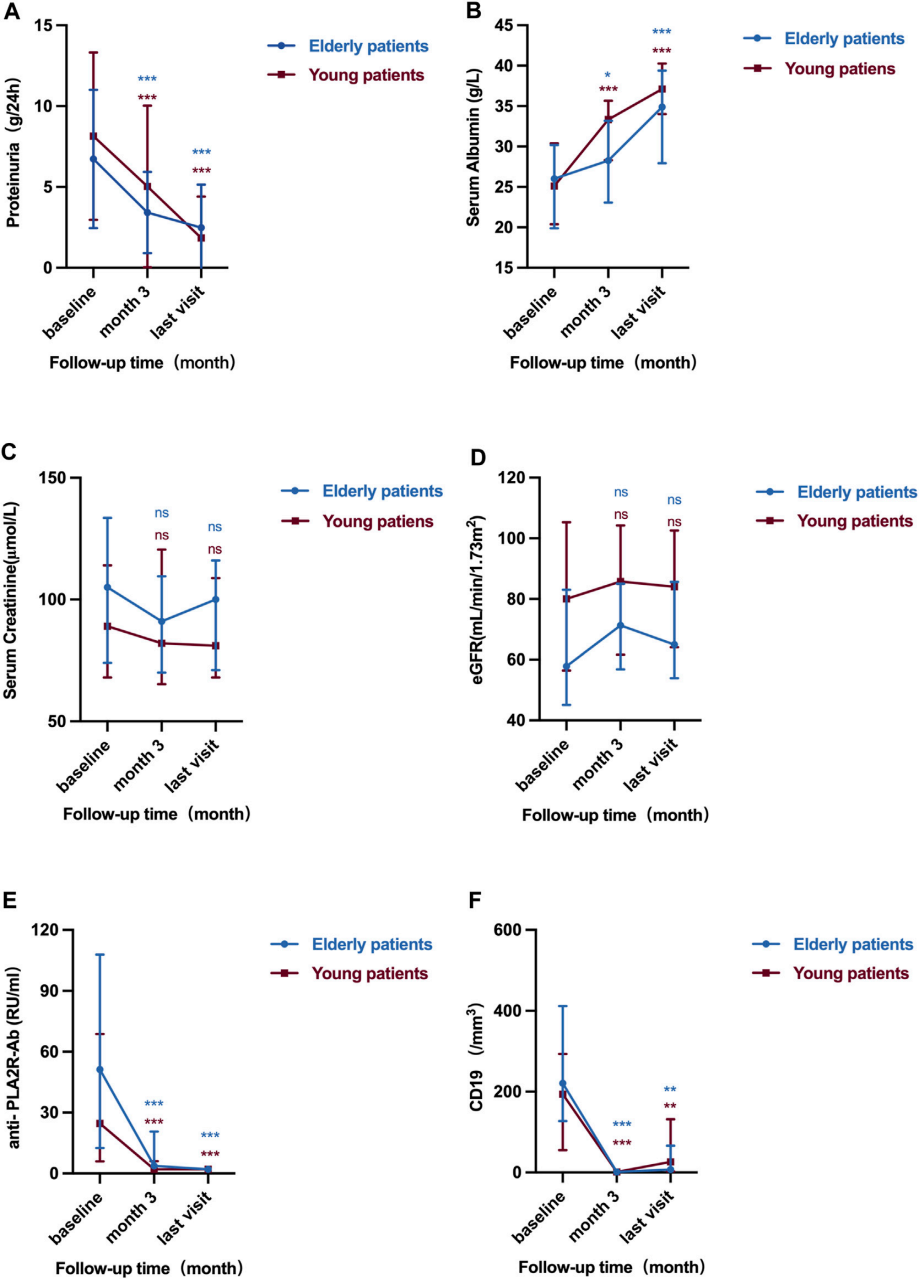
Serial levels of 24 h urinary protein **(A)**, serum albumin **(B)**, serum creatinine **(C)**, eGFR **(D)**, anti-PLA2R antibody **(E)** and CD19^+^ B cell counts **(F)** after the rituximab treatment in elderly patients and young patients. The bars show median with interquartile range for each variable. ns means *p* > 0.05 vs. baseline; *p < 0.05 vs. baseline; **p < 0.01 vs. baseline; ***p < 0.001 vs. baseline.

In addition to CD19^+^ B cell counts and anti-PLA2R antibody, there were also no differences in the level of proteinuria, serum albumin, creatinine, and eGFR at month 3 and last visit after RTX treatment between elderly patients and young patients (all *p* > 0.05). The results showed that in both elderly patients and young patients, improvements in proteinuria and serum albumin were generally occurred from baseline to month 3 and were continued to improve throughout the study. Levels of proteinuria in elderly patients decreased from 5.04 (3.98, 9.22) g/d to 2.87 (1.32, 5.79) g/d at month 3 and to 1.60 (0.49, 3.75) g/d at the last visit (all *p* < 0.001). Consistent with the results of proteinuria, after 3 months of RTX treatment, compared with the baseline levels, the level of serum albumin in elderly patients increased significantly [27.25 (23.08, 33.18) vs. 21.40 (16.75, 25.00) g/L, *p* = 0.027]. At the end of follow-up, patients were further improved as evidenced by significant improvements in serum albumin [34.90 (28.10, 39.05) vs. 21.40 (16.75, 25.00) g/L, *p* < 0.001]. The renal function in elderly patients stayed stable with eGFR from 57.84 (46.33, 81.93) mL/min/1.73 m^2^ to 68.45 (51.51, 86.76) mL/min/1.73 m^2^ and 64.95 (54.10, 85.42) mL/min/1.73 m^2^ at month 3 and last visit (Table 2) (Figure 2).

### 3.3 Factors associated with nonremission

In order to explore the critical factors predicting the therapeutic effects of RTX in MN patients and evaluate whether advanced age is an independent risk factor that influence the response to RTX treatment, we conducted regression analysis. Baseline characteristics were used as independent variables, and the outcomes of MN was used as the dependent variable for the logistic regression. The results of multivariant regression analysis showed that for each increase of 1 g/L in serum albumin, the risk of nonremission would decrease by 29.0% in MN patients treated with RTX (OR = 0.710, 95% CI 0.514–0.981, *p* = 0.038) (Table 3).

**TABLE 3:** Univariable and multivariable model results for the risk of nonremission at last visit using the baseline characteristics.

Variable	Univariate analysis		Multivariate analysis	
	OR (95%CI)	*p*-valve	OR (95%CI)	*p*-valve
Female sex	1.800 (0.556, 5.829)	0.327	13.521 (0.073, 218.01)	0.329
Age at the first infusion (years)	1.040 (0.980, 1.080)	0.181	1.595 (0.843, 3.020)	0.151
BMI (kg/m^2^)	1.027 (0.914, 1.155)	0.653	0.630 (0.358, 1.109)	0.109
Hemoglobin (g/L)	0.979 (0.956, 1.003)	0.087	1.061 (0979, 1.149)	0.152
Serum creatinine (µmol/L)	1.008 (1.001, 1.015)	0.054	0.997 (0.976, 1.018)	0.772
24 h urinary protein (g/d)	0.990 (0.898, 1.090)	0.832	1.289 (0.892, 1.861)	0.176
Albumin (g/L)	0.871 (0.795, 0.954)	0.003^a^	0.710 (0.514, 0.981)	0.038^a^
anti-PLA2R-Ab titer (RU/mL)	1.005 (1.002, 1.008)	0.003^a^	1.001 (0.994, 1.008)	0.746
Rituximab dose (g)	0.964 (0.553, 1.680)	0.897	0.192 (0.015, 2.471)	0.206
Median time since biopsy-proven diagnosis (months)	0.868 (0.734, 1.027)	0.098	0.817 (0.528, 1.266)	0.366

^a^ Stands for *p* < 0.05. OR, odds ratio; CI, confidence interval; BMI, body mass index; anti- PLA2R-Ab, anti-phospholipase A2 receptor antibody.

### 3.4 Safety analysis of RTX used in elderly patients with MN

In order to explore the risk factors of serious infections of in MN patients treated with RTX and evaluate whether advanced age is an independent risk factor of serious infections in MN patients treated with RTX, we conducted regression analysis. The results of multivariant regression analysis showed that for each increase of 1 g/L in serum albumin, the risk of serious infection would decrease by 43.2% (OR = 0.568, 95% CI 0.334–0.969, *p* = 0.038). Elderly patients have an increased risk of serious infection, compared with patients younger than 70 years (OR = 32.874, 95% CI 1.300–831.490, *p* = 0.034) (Table 4).

**TABLE 4:** Univariable and multivariable model results for the risk of a serious infection at last visit using the baseline characteristics.

Variable	Univariate analysis		Multivariate analysis	
	OR (95%CI)	*p*-valve	OR (95%CI)	*p*-valve
Female sex	2.260 (0.261, 19.588)	0.459	0.599 (0.022, 16.204)	0.183
Age≥70 years old	0.069 (0.008, 0.596)	0.015^a^	32.874 (1.300, 831.490)	0.034^a^
BMI (kg/m^2^)	0.813 (0.630, 1.049)	0.112	0.650 (0.345, 1.225)	0.183
Hemoglobin (g/L)	0.987 (0.950, 1.025)	0.484	0.982 (0.911, 1.058)	0.633
Serum creatinine (µmol/L)	1.003 (0.992, 1.014)	0.597	0.986 (0.962, 1.011)	0.285
24 h urinary protein (g/d)	0.885 (0.707, 1.107)	0.283	0.168 (0.025, 1.149)	0.069
Albumin (g/L)	0.887 (0.758, 1.015)	0.079	0.568 (0.334, 0.969)	0.038^a^
CD4 count (/mm^3^)	0.999 (0.997, 1.001)	0.486	1.001 (0.997, 1.006)	0.568
CD19 count (/mm^3^)	0.999 (0.993, 1.004)	0.576	1.000 (0.992, 1.008)	0.981
Median time since biopsy-proven diagnosis (months)	1.020 (0.996, 1.045)	0.105	1.030 (0.992, 1.070)	0.121
Median time since discontinuation of prior immunosuppressants (months)	0.967 (0.919, 1.017)	0.192	1.016 (0.921, 1.121)	0.752
anti-PLA2R-Ab titer (RU/mL)	1.000 (0.994, 1.005)	0.864	0.987 (0.971, 1.003)	0.104
Rituximab dose (g)	0.458 (0.137, 1.535)	0.206	0.077 (0.004, 1.476)	0.089

^a^ Stands for *p* < 0.05. OR, odds ratio; CI, confidence interval; BMI, body mass index; anti- PLA2R-Ab, anti-phospholipase A2 receptor antibody; CD, cluster of differentiation.

Among the 37 elderly patients treated with RTX, 6 developed pneumonia. Of these 6 patients, 3 were cured after anti-infective treatment, while the other 3 patients failed to respond to treatment and eventually died (Table 5). Organisms were detected by metagenomic next-generation sequencing using bronchial alveolar lavage fluid in two patients who were decreased. *Candida* albicans and *Streptococcus* constellans were detected in one patient’s bronchial alveolar lavage fluid. Pneumocystis jirovecii and Aspergillus flavus were detected in another patient’s bronchial alveolar lavage fluid. No pathogenic bacteria were detected in the sputum of the third decreased patient by conventional tests. This result also demonstrated that pulmonary infections are common in elderly patients with MN undergoing RTX treatment.

**TABLE 5:** Adverse events in elderly patients and young patients receiving rituximab.

Event	Age ≥ 70 years (*n* = 37)	Age < 70 years (*n* = 76)	*p*-valve
	Patients, n (%)	Patients, n (%)	
Any adverse event	12 (32.43%)	17 (22.37%)	0.250
Serious adverse events	7 (18.92%)	4 (5.26%)	0.022^a^
Fatal	3 (8.11%)	0 (0.00%)	0.012^a^
Nonfatal	4 (10.81%)	4 (5.26%)	0.281
Pneumonia	3 (8.11%)	1 (1.32%)	0.067
Upper respiratory tract infection	1 (2.70%)	3 (3.95%)	0.747
Nonserious adverse events	5 (13.51%)	13 (17.11%)	0.624
Infusion reactions	3 (8.11%)	10 (13.16%)	0.430
Leukopenia	2 (5.41%)	3 (3.95%)	0.724

^a^ Stands for *p* < 0.05. *p* values are for the difference in proportions of patients having a specific type of event.

## 4 Discussion

MN is the most common primary nephrotic syndrome in elderly patients, with a higher incidence than young people. Studies had shown that elderly patients have a lower eGFR, a higher incidence of hypertension, a higher proportion of glomerulosclerosis, more severe renal tubular atrophy and interstitial fibrosis, and a higher risk of progression to end stage renal disease compared to young patients. Furthermore, elderly patients have a higher risk of infections and consequent mortality especially if with immunosuppressive drug therapies (Bae et al., 2018). Therefore, choosing a reasonable and effective treatment plan is particularly vital for elderly patients with MN.

This retrospective single center study demonstrated that RTX is effective for remission induction in elderly patient with MN. This study demonstrated that 18.92% and 70.27% of patients achieved complete remission and complete or partial remission within a median follow-up time of 15.50 months, which is similar to previously reported response rates of approximately 50%–70% (Teisseyre et al., 2021; Ou et al., 2022). However, through this study, we also found that pulmonary infections seemed to be common in those patients.

RTX is a human-mouse chimeric monoclonal antibody that specifically targets the surface antigen CD20 of B cells, which is a transmembrane phosphoprotein expressed on the membrane surface of early B cells (Salles et al., 2017). RTX binds to CD20 and consumes CD20+B cells through three ways: antibody dependent cell mediated cytotoxicity, complement dependent cytotoxicity, and direct induction of cell apoptosis (Smith, 2003). In recent years, RTX had gradually been used for the treatment of MN, and its therapeutic mechanism may be to reduce the level of B cells, reduce the production of circulating antibodies, and prevent the formation of subcutaneous immune deposits in the glomerulus, reduce the damage to the glomerular filtration barrier, and thus alleviate MN (Kim et al., 2019). Due to the lack of CD20 antigen expression in hematopoietic stem cells, normal plasma cells, or other normal tissues, RTX selectively reduces B lymphocytes, which can inhibit the production of autoantibodies and does not have non-specific immunosuppressive toxicity (Smith, 2003). Since 2002, based on various clinical studies of RTX treatment for MN and 2021 KDIGO recommendations, compared to traditional treatment regimens, RTX treatment for MN had been fully recognized in clinical applications, both in terms of efficacy and safety (Fervenza et al., 2019; Scolari et al., 2021; Dahan et al., 2017; Kidney Disease: Improving Global Outcomes KDIGO Glomerular Diseases Work Group, 2021).

There are currently few studies evaluating the efficacy and tolerability of RTX in the treatment of the elderly population. A prospective cohort study included 191 patients with rheumatoid arthritis who received RTX treatment. Among them, 26.5% of patients aged 75 and above reported severe infections, with a higher incidence than younger patients (Payet et al., 2014). A retrospective study included 31 patients aged 60 and above with ANCA associated vasculitis who received RTX, and the results showed that RTX could induce remission. However, 16% of patients developed severe infections within the first year (Timlin et al., 2015). A multicenter cohort study published in 2022 included 93 patients aged 75 and older with ANCA associated vasculitis. The study found that RTX combined with high-dose glucocorticoids as induction therapy was associated with a higher incidence of severe infection and mortality (Thietart et al., 2022). However, it is still unknown whether RTX is a safe and effective treatment regimen in elderly patients with MN. To our knowledge, the current study is the first one to evaluate the outcomes and adverse events associated with the use of RTX in the treatment of elderly patients with MN.

This study showed that in MN patients with advanced age appeared to be more susceptible to pulmonary infection. Currently, there are no studies to evaluate whether advanced age is an independent risk factor for infection in MN patients receiving RTX treatment. However, there are studies that confirm age itself is a risk factor for serious infections among patients with MN, even when those patients receive treatment with drugs other than rituximab. In a cohort study of 135 patients with MN who had a median age of 67 years and were followed up for 4 years, the results showed that older age was a robust predictor of infection (adjusted OR, 5.27; 95% CI, 1.31–21.20) (Kim et al., 2019). Two mechanisms are assumed to increase the rate of serious infections. The first is a reduction in immunity due to aging. Age-associated immunosenescence leads to reduced ability to resist infection, and infection leads to increased damage and loss of homeostasis which in turn leads to further accelerating immunosenescence (Borgoni et al., 2021; Santoro et al., 2021). The second might be the number of comorbidities. Our data showed an increased incidence of diabetes mellitus and hypertension in elderly patients. However, these differences were not statistically significant, which may be due to the small sample size. Our study also found that hypoalbuminemia was an independent risk factor for serious infections in MN patients treated with RTX. Similar to our study, the results of a cohort study of 333 MN patients treated with cyclosporin showed that lower plasma albumin level was a risk factor of pulmonary infection (Wang et al., 2019). In MN patients, hypoalbuminemia is not only associated with large amounts of proteinuria, but may also be associated with malnutrition. The possible mechanisms of infection associated with hypoalbuminemia are as follows. Hypoalbuminemia is associated with the acquisition and severity of viral, bacterial, and fungal infections and predicts infectious complications in non-infective disease. Hypoalbuminemia is associated with the acquisition and severity of infectious diseases due to the fact that the intact innate and adaptive immune response is largely dependent on albumin. Oxidation and catabolism of albumin affect interactions with biologically active lipid mediators that play an important role in antimicrobial defense. A causal relationship between hypoproteinemia and increased risk of infections is biologically plausible (Wiedermann, 2021).

The study has some limitations. Firstly, this study was limited by its retrospective design. Secondly, the number of patients included in the study was relatively small. Finally, the study lacked a comparison with the control group such as elderly patients who received cyclophosphamide or calcineurin inhibitor. In future studies, larger sample studies are needed to evaluate the efficacy and safety of RTX in elderly patients with MN, and future efforts might focus on finding the best regimen to reduce infections without limiting benefits in this population.

## Data Availability

The raw data supporting the conclusion of this article will be made available by the authors, without undue reservation.
